# Image Analysis to Estimate Mulch Residue in Soil

**DOI:** 10.1155/2014/617408

**Published:** 2014-09-17

**Authors:** Carmen Moreno, Ignacio Mancebo, Antonio Saa, Marta M. Moreno

**Affiliations:** ^1^Universidad de Castilla-La Mancha/Escuela de Ingenieros Agrónomos, Ronda de Calatrava 7, 13071 Ciudad Real, Spain; ^2^Departamento de Edafología y Climatología, ETSI Agrónomos, Universidad Politécnica de Madrid, Ciudad Universitaria sn, 28040 Madrid, Spain; ^3^CEIGRAM, Universidad Politécnica de Madrid, Ciudad Universitaria sn, 28040 Madrid, Spain

## Abstract

Mulching is used to improve the condition of agricultural soils by covering the soil with different materials, mainly black polyethylene (PE). However, problems derived from its use are how to remove it from the field and, in the case of it remaining in the soil, the possible effects on it. One possible solution is to use biodegradable plastic (BD) or paper (PP), as mulch, which could present an alternative, reducing nonrecyclable waste and decreasing the environmental pollution associated with it. Determination of mulch residues in the ground is one of the basic requirements to estimate the potential of each material to degrade. This study has the goal of evaluating the residue of several mulch materials over a crop campaign in Central Spain through image analysis. Color images were acquired under similar lighting conditions at the experimental field. Different thresholding methods were applied to binarize the histogram values of the image saturation plane in order to show the best contrast between soil and mulch. Then the percentage of white pixels (i.e., soil area) was used to calculate the mulch deterioration. A comparison of thresholding methods and the different mulch materials based on percentage of bare soil area obtained is shown.

## 1. Introduction

Mulching is a method of improving the condition of agricultural soils by covering the soil surface with different kinds of materials. For this purpose, black polyethylene (PE), a petroleum-based plastic, is the most used due to its low price and the positive effects on crop yields [1, 2]. However, a major problem derived from its use is how to remove it from the field and how it can be completely done [2], because the useful life of plastic materials exceeds the duration of crop cycles, and they are usually left in the soil afterward. Although the part exposed to the light undergoes photodegradation and favors the plastic decomposition, the rest of the material is broken into pieces by ploughing labors, some pieces being buried or remaining on the soil surface. The buried pieces are more difficult to decompose since they are less affected by light and heat, creating serious soil problems whose environmental repercussion has not been fully evaluated [3]. Feuilloley et al. [4] found that it is difficult to foresee the accumulative effects of PE fragments and the impact of the repeated use of these PE films on the environment. In this context, the microfragments derived from the buried pieces are electrically charged and their impact, if accumulated, on the argilo-humic complex is unknown. With the aim of accelerating the PE fragmentation in the soil, special prooxidant additives are used. These substances contain different complexes of transition metals, particularly Fe, Co, and Mn [5], with the environmental risk associated with soil heavy metal accumulation.

One possible solution to these environmental problems is to use biodegradable plastic or paper, as mulch, which could present an alternative to polyethylene in reducing nonrecyclable waste and decreasing the environmental pollution associated with it [6–8]. On the other hand, it is essential to control the functionality of these materials in the soil. This can be done at the laboratory, such as by measuring the transmittance of the materials [9], which would be quite complex, or at field level, by determining the level of deterioration of materials. In the latter case, it is very common to use qualitative scales to measure the degree of disrepair, for example, 9 or no deterioration (material practically intact) down to 1 or totally degraded, as indicated by Martin-Closas et al. [10], accompanied by photographic monitoring, apart from visual inspection, which is largely subjective.

Determination of mulch residues on the ground is one of the basic requirements to estimate the potential of each material to degrade. Determining the extent of mulch residue in the field is an exhausting job and there is not a distinct and accurate criterion for its measurement, as reported in detail in the review by Cowan [11]. There are several indices to estimate the residue covers but most of them are not only laborious and time consuming but also greatly affected by human errors. Human vision is fast and accurate enough in this case but the problem is that the magnitude must be stated numerically to be reported and to be used for comparison between different mulches or mulches at different times. Interpretation of the extent perceived by vision into figures is possible by a simulation of the human vision system.

Machine vision comprising image processing systems can perform these tasks. Lately, recent developments in robotics, computer vision, and hardware have helped to solve several problems in agriculture. Thus, the information provided by digital imaging has been used extensively in ground classification cover [12], leaf area index estimation in forest ecosystems [13], identifying eroded areas [14], mechanical properties of horticultural products [15], crop classification [16], and weed recognition [17]. In these latter cases, it is important to have efficient and automatic image segmentation to distinguish vegetation from the ground (Guijarro et al. [18] and references therein).

**Figure 1:**
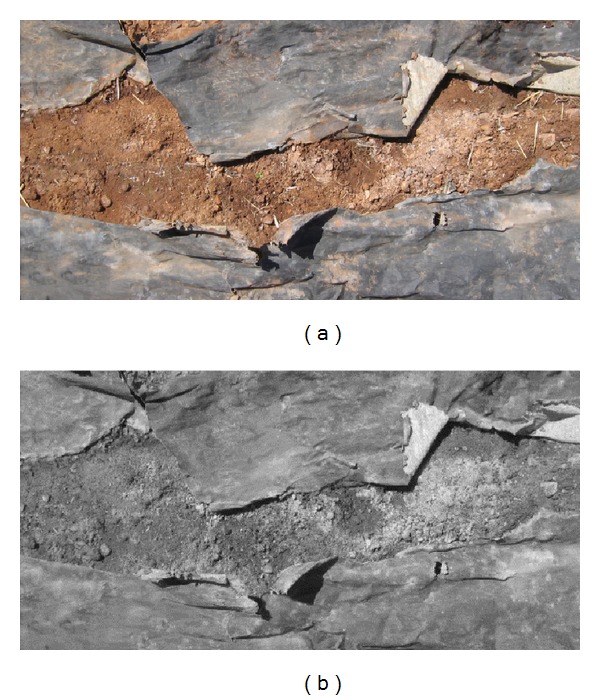
Image with mulch and soil 100 days after field implementation. (a) Original image in RGB color space; (b) grayscale image.

However, these images in field conditions, with soil and mulch, are quite complex, especially because of the small contrast between them. Meanwhile the initial appearance of most of the mulches is a kind of black PE and at the end of the crop campaign the materials appeared somewhat discolored and soil and/or crop residue was impregnated making it very difficult to completely remove them (Figure 1(a) as an example).

This work has a double objective: first, to determine an image analysis method, reliable and accurate, in order to evaluate the residue of the mulch materials at the end of the crop season, and based on it, their deterioration level; and second, to determine and to compare the mulch residual of alternative materials to polyethylene. In order to achieve this, four thresholding methods were applied to binarize the images of four different mulch materials. Then a simple matrix calculation automatically determined the soil area as a measure of mulch deterioration. The results are compared with an area obtained by an expert user of imaging software. A preliminary idea of this work can be consulted in Moreno et al. [19].

## 2. Material and Methods

### 2.1. Mulch Materials and Image Acquisition

The mulch materials used were two black biodegradable plastics (BD) composed of corn starch (mulch 1: 15 *μ*m, Novamont; mulch 2: 17 *μ*m, Barbier), standard black polyethylene (PE) (mulch 3: 15 *μ*m, Siberline), and one black paper (PP) (mulch 4: 85 g m^−2^, Mimcord).

Images were taken 100 days after the mulch was implanted in the ground to determine the deterioration, in August 2009. During the experimental period, meteorological data such as mean, mean maximum, and mean minimum temperatures, rainfall, and solar radiation were 22.8°C, 31.8°C, 12.3°C, 83.0 mm, and 27.1 MJ m^−2^, respectively. The corresponding mean values for the previous 9-year historical series (2000–2008) were 19.6°C, 27.2°C, 10.9°C, 49.0 mm, and 25.9 MJ m^−2^, respectively. Irrigation was applied, so the soil had moisture conditions similar to those reached with summer vegetable crops. Also the time spent on the ground was adjusted to the duration of a horticulture crop cycle (e.g., tomatoes and peppers). The test was carried out in the experimental farm “El Chaparrillo” (3°56′W–39°0′N, altitude 640 m), property of the Junta de Comunidades de Castilla-La Mancha, in Ciudad Real (Central Spain).

A digital camera (Canon PowerShot A80-35 mm) was used to acquire color digital images (JPG format) under similar lighting conditions (sunny day and at noon) at the experimental field. A total of 24 photographs, 6 per mulch, were taken according to a randomized block design. Images were captured accurately covering a 1 × 0.5 meter frame which yielded images cropped to be 2400 × 1200 pixels. The images were processed with the Image Processing Toolbox from Matlab R2009a [20] belonging to The Mathworks, Inc. (Natick, MA, USA).

### 2.2. RGB and HSV Color Space Histograms

“Color” refers to the human brain's subjective interpretation of combinations of a narrow band of wavelengths of light. González and Wood [21] define the “color space” transformations as various specifications of a three-dimensional coordinate system where each color is represented by a single point. The RGB model (red, green, and blue) is based on a system of Cartesian coordinates where each point is described by its primary red, green, and blue spectral components. RGB image color consists of three independent image planes, one for each primary color. Another color space is HSV, where each point is defined by hue (H), saturation (S), and value (V) coordinates.

The HSV color space has a better capability of representing the colors of human perception than the RGB color space does. The H and S components are closely related to the human eye color perception. The third component (V) is related to image brightness [21]. The transformation equations from RGB to HSV space are commonly used and can be consulted in the work of Chun-Ming Tsai [22]. The use of different color spaces is applied in agriculture, especially in plant detection, to discern the plant from the background [23, 24].

Both spaces (RGB and HSV) have been taken into consideration to study the image histograms derived from them and to see which one was better to binarize the image.

### 2.3. Image Segmentation

The segmentation process partitions the digital image into disjoint regions [25], the automated segmentation being, in general, one of the most difficult tasks in the image analysis [26]. There are many color segmentation techniques reported in the literature, such as texture analysis, edge detection, region split and merging, feature analysis and histogram thresholding or clustering, the last being the most commonly used, as indicated by Du et al. [27].

Image segmentation by the thresholding technique involves the assumption that the objects and the background have distinct level distributions and so the histogram contains two—or more—distinct peaks and the threshold value separating them can be obtained. If the histogram is bimodal, the image can be segmented into two classes or regions: the object with value levels above the threshold (*t*) and background with values below the threshold, or vice versa [21, 28]. Then, usually, a binary (black and white) thresholded *B*(*x*, *y*) image is obtained from the corresponding histogram image *f*(*x*, *y*): a value of 1 is assigned to the pixels of the object, and 0 to the background pixels. A survey on threshold selection techniques can be consulted at Sahoo et al. [28]. This study identified the soil as foreground and mulch as background.

When the threshold depends only on *f*(*x*, *y*), the threshold is called global, while if it also depends on local properties of each point (*x*, *y*), the threshold is called local. In this study we have two global methods and one local method. The global Otsu [29] (OT) and Ridler-Calvard [30] (RC) thresholds have been chosen because they are widely used thresholding techniques which have proven their effectiveness in various fields [31]. Furthermore, the OT method is implemented as the default approach to image thresholding in some free or commercial software such as Matlab. The local entropy-based thresholding method (LE) is a local thresholding method that has been shown to be promising and effective in image thresholding [27, 32]. Below we briefly explain each method.

Otsu's method (OT) [29] has shown great success in image segmentation. Several improved versions of Otsu's method have been proposed, such as a recursive Otsu's method by Cheriet et al. [33] or the version of the method given by Xu et al. [34].

OT finds the threshold (*t*) that maximizes the between-class variance (background and foreground), *σ*_*b*_^2^, in the image histogram. Equivalently, OT finds the threshold (*t*) that minimizes the within-class variance, *σ*_*w*_^2^.

The principle of the RC method [30, 35] is to evaluate the threshold (*t*) for any image with a bimodal histogram by assuming *t* to be *t* = (*m*_1_ + *m*_2_)/2, where *m*_1_ and *m*_2_ are the means of each of the two components of the histogram separated by the threshold. For this, an initial threshold is selected, and a new threshold is obtained by averaging the means of the two classes. The process continues until the value of the threshold converges. When the iterative algorithm stops, the threshold calculated is the average of the mean levels of the two classes [34, 36].

LE thresholding is based on the maximization of the information measure between two classes, foreground and background. Therefore, the optimal *t* maximizes the addition of foreground and background entropy [37–39]. A survey and comparative analysis of entropy and relative entropy thresholding techniques can be consulted in Chang et al. [40].

Entropy refers to the amount of information that can be obtained from a set of messages and was first introduced into information theory by Shannon [41]. The entropy of an image can be defined as *H* = −∑_*i*=1_^*i*=*L*^ *p*_*i*_log⁡_2_⁡*p*_*i*_, where *p*_*i*_ is the probability that the gray-value *i* appears in the image, and *L* is the maximum gray-value.

Additionally, threshold setting by the user, partly subjective, in the image histogram is quite frequently applied and therefore also incorporated into the study named as manual thresholding (MT).

### 2.4. Performance to Obtain Binary Images and Percentage of Bare Soil Area

The thresholding methods tested (Otsu, Ridler-Calvard, local entropy, and manual thresholding) were applied to the histograms corresponding to each independent plane (red, blue, and green at RGB color space and hue, saturation, and value at HSV color space).

Otsu's method was applied directly using Matlab commands; Ridler-Calvard method was implemented automatically using the iterative Isodata algorithm [20], and the development of the local entropy method is based on Du et al. [27] and Du [32] works.

The result of segmentation using the proposed methods is a binary image (*B*′) with white pixels representing bare soil and black pixels representing the mulch. A last step consisted of applying to each *B*′ binary image a morphological operation to reduce the noise regions with an area smaller than *m* × *n*/100. Removing small objects (both in foreground and background) was carried out by opening the binary areas (in both binary image *B*′ and its complement) with an 8-connectivity. Then all small objects were removed to obtain the final binary image (*B*). This operation was performed using a specific Matlab function.

The area estimated was the percentage of bare soil and it is determined by dividing the number of white pixels by that of all pixels of the image.

Manual thresholding was based on the image histogram. Most image processing software programmes, including Matlab or Photoshop, have an interactive contrast and brightness adjustment tool that can be associated with a grayscale image. Then the thresholds were adjusted in the histograms to achieve the best discrimination between soil and mulch.

The reference areas (*A*_*R*_) were obtained from the color images to use them as the optimum classification. They were obtained by an expert user of Adobe Photoshop CS 3 software who used a zoom of 800 to discriminate soil and mulch.

### 2.5. Statistical Analysis

A paired 2-tailed Student's* t*-test was performed to determine the variability in the metrics for each pair of areas: area of reference *A*_*R*_ (obtained by the expert of Adobe Photoshop CS 3) and area using a threshold method. A paired 2-tailed Wilcoxon nonparametric test was used in case of no normality (tested by Shapiro-Wilk's test).

An analysis of variance (ANOVA) was applied to compare the degradation among the mulch materials, by comparing the percentage of soil area estimated by each thresholding method.

## 3. Results and Discussion

### 3.1. Histograms Selected

**Figure 2:**
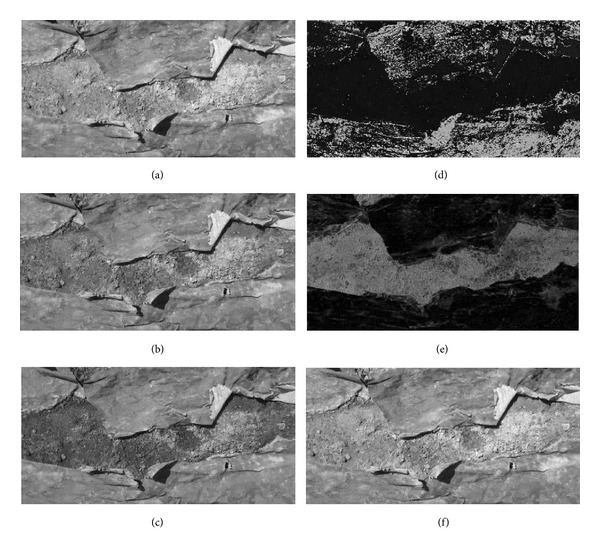
Representation of the original image shown in Figure 1 in the RGB system (left column) and in the HSV system (right column). (a) Red plane, (b) green plane, (c) blue plane, (d) hue plane, (e) saturation plane, and (f) value plane.

The original RGB images used in this work had the problem of presenting a very low reflectance difference between soil and mulch, with low contrast also in each of the three separate color planes (Figures 2(a), 2(b), and 2(c)).

**Figure 3:**
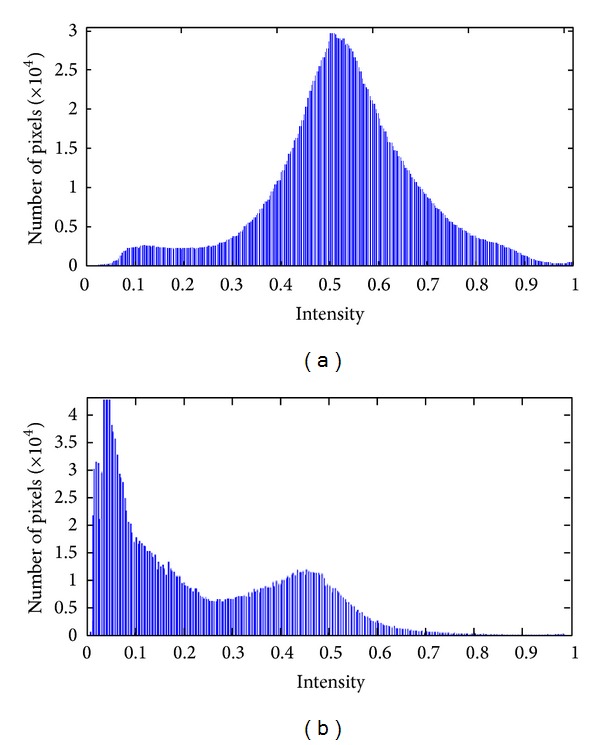
Histograms corresponding to color image of Figure 1 in (a) grayscale image and (b) saturation plane.

The histograms of the grayscale image corresponding to the original RGB color images were practically unimodal (Figure 1(b) and its histogram in Figure 3(a)); that is, soil and mulch looked confused to each other. Neither of the images of *R*, *G*, and *B* obtained from the separate RGB color planes showed good contrast levels (Figures 2(a), 2(b), and 2(c)). For this reason we carried out the conversion of images from the RGB color space to HSV space and the three independent planes (Figures 2(d), 2(e), and 2(f)) were examined.

All images corresponding to saturation plane provided a good contrast between soil and mulch (Figure 2(e)) and the histograms appeared bimodal (Figure 3(b)). So these histograms were chosen to give the corresponding binary images by applying the four thresholding methods.

### 3.2. Comparison of Thresholding Methods and Computational Time

In all images it was fulfilled that the relationship between the Ridler-Calvard and the Otsu thresholds was *t*_RC_ < *t*_OT_ (Table 1). Therefore, in the binary images obtained initially (prior to performing morphological operations), the relationship between the values corresponding to the bare soil (white) obtained through these thresholds was the opposite: *A*_OT_ < *A*_RC_. Additionally, in most of the images the following was satisfied: *t*_MT_ < *t*_RC_ < *t*_OT_, and therefore the ratio of initial areas (data not shown) was *A*_OT_ < *A*_RC_ < *A*_MT_ for all of them. In these cases, the area corresponding to bare soil was overestimated by the *t*_MT_ threshold, especially when compared with *t*_OT_. Guijarro et al. [18] also observed that the threshold obtained by Otsu's method tended to produce an undersegmentation of white pixels, corresponding to barley and corn crops, because it provided a relatively high value in the histogram.

For example, the thresholds corresponding to Figure 1 were *t*_MT_ = 0.2499, *t*_RC_ = 0.2677, and *t*_OT_ = 0.2745, and the corresponding bare soil areas, prior to performing morphological operations (expressed as a percentage), were 39.16, 36.41 and 35.78, respectively.

The maximum difference between the *t*_OT_ − *t*_RC_ thresholds was 0.0191, while the maximum differences between abs(*t*_OT_ − *t*_MT_) and abs(*t*_RC_ − *t*_MT_) were higher (0.2350 and 0.2541, resp.). The averages of the differences among abs(*t*_OT_ − *t*_RC_), abs(*t*_OT_ − *t*_MT_), and abs(*t*_RC_ − *t*_MT_) were 0.0046, 0.0530, and 0.0515, respectively. All this proves the close proximity existing between the *t*_OT_ and *t*_RC_ thresholds and the greater difference they show in relation to the manual threshold.

Xu et al. [34] proved that the optimal Otsu (*t*) threshold is equal to the average value of the mean levels of two classes partitioned by this threshold. This result revealed the Ridler-Calvard method as an iterative version of Otsu's method, and therefore both approaches to image thresholding would be very close [31]. However, the slight right shift of the *t*_OT_ threshold observed in the histograms would indicate, according to the studies by Xu et al. [34] and Xue et al. [31], that the class with pixels of bare soil (foreground) has larger variance than the class with pixels of mulch (background). According to these studies, *t*_OT_ tends to balance the two classes, deviating from the intersection point of the two classes toward the class with larger variance. Also, *t*_OT_ shifts to the bigger size class when the size difference between background and object is very significant. This occurs in pictures 17 and 18 (Table 1), in which the size of the foreground class is much smaller than the background class, so *t*_OT_ would shift left from the histogram valley. These findings [31, 34] could explain the infrasegmentation concerning the identification of green obtained by Guijarro et al. [18] with the Otsu threshold.

The thresholds obtained by the LE method did not follow a pattern of behavior similar to the thresholds obtained by the other methods. The *t*_LE_ thresholds were very different from the *t*_OT_ and *t*_RC_. Du et al. [27] also obtained OT and LE thresholds very different from each other in the *R*, *G*, and *B* color domains. However, when comparing four gray level thresholding methods (Otsu, Pal and Pal's local entropy, joint entropy, and the joint relative entropy methods), no conclusions could be drawn on which thresholding method performed better than the others. In this context, Glasbey [42], when comparing bimodal histograms for 11 thresholding methods including Otsu, Ridler-Calvard, and other methods based on entropy, found that the entropy method generated the widest spread of thresholds.

The initial binary images obtained from OT, RC, and MT thresholding methods differed little from the final binary images (after eliminating the small objects), both visually and in terms of the value of the area. For example, the respective areas before and after eliminating the small objects in the image presented in Figure 1 were 39.1596 and 38.2370 for MT, 36.4105 and 35.7161 for RC, and 35.7760 and 36.4270 for OT thresholding methods. In the case of the binary images obtained by the LE method, there were higher visual differences before and after performing morphological operations (for the cited image, the areas were 51.7199 and 57.5681, resp.).

**Figure 4:**
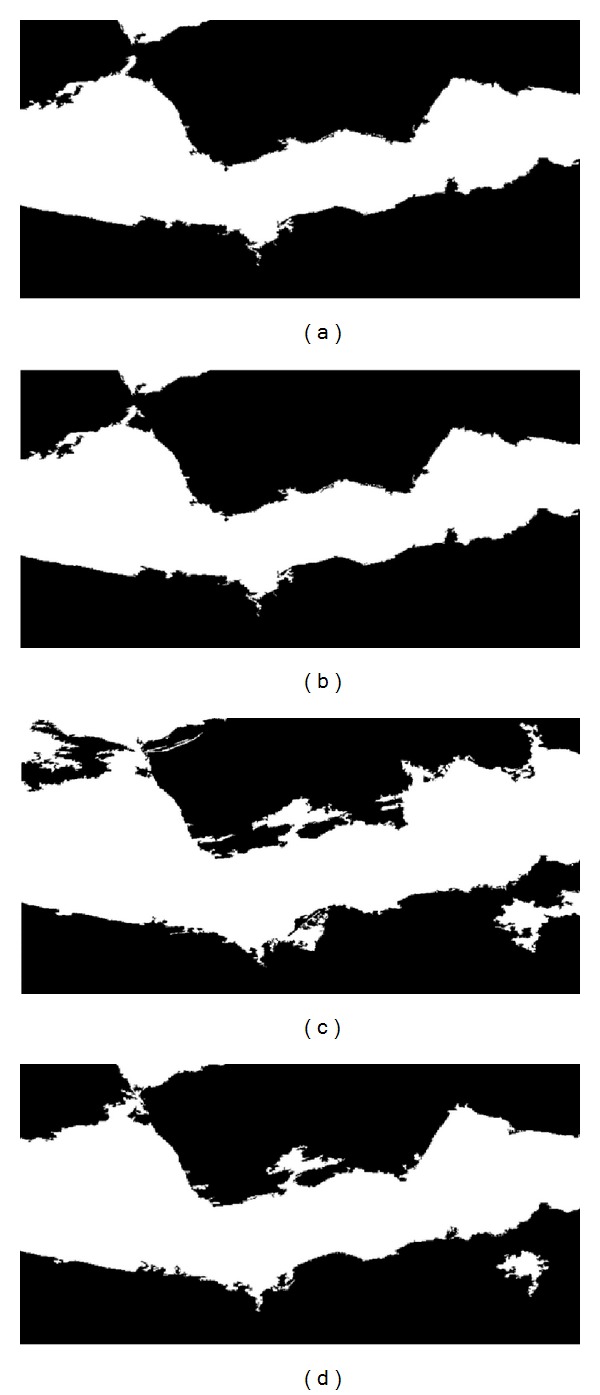
Binary images from saturation plane (HSV color system), obtained from the original image showed in Figure 1, applying different thresholding methods: (a) Otsu (*t*_OT_ = 0.2745; *A*_OT_ = 36.43); (b) Ridler-Calvard (*t*_RC_ = 0.2677; *A*_RC_ = 35.72); (c) local entropy (*t*_LE_ = 0.1412; *A*_LE_ = 57.57); (d) manual thresholding (*t*_MT_ = 0.2499; *A*_MT_ = 38.24). Black pixels represent mulch and white pixels represent bare soil.

In general, the binary images derived from the OT and RC thresholds gave a better visual fit to reality than those derived from both the manual and the LE thresholds (Figure 4 as an example).

Moreover, the time spent in obtaining areas using a manual threshold was higher than the automatic thresholding methods, as expected (Table 2). Among the automatic methods, LE gave a longer computational time, in fact double the time of the RC. Calculating the ratio with respect to the MT method, the automatic methods from faster to slower are RC, OT, and LE (Table 2). In accordance with Glasbey [42], RC is slightly less computationally intensive than Otsu.

### 3.3. Comparison among Bare Soil Areas

The comparison of the areas obtained from each method with the reference area (*A*_*R*_) is shown in Table 3. There we can see the results of the paired 2-tailed Student's *t*-test between the *A*_*R*_ and the area obtained by the OT, RC, and MT thresholding methods (*A*_OT_, *A*_RC_, and *A*_MT_, resp.). In the comparison of the area provided by LE (*A*_LE_) and *A*_*R*_, due to the lack of normality (shown by the Shapiro-Wilk's test) in the differences of the corresponding pairs of areas, the Wilcoxon test (for paired data) was performed. Statistically significant differences (*P* < 0.05) between *A*_*R*_ and *A*_MT_ were obtained; however, the visual appearance of both images was quite similar.

Despite the dubious significance of the paired 2-tailed Wilcoxon test for *A*_*R*_ and *A*_LE_ areas, the differences between these values became important (Table 1), with max abs(*A*_*R*_ − *A*_LE_) = 40.1930. In these cases, visual differences between both types of binary image were perceived. The average of these differences, in absolute value, was 18.01, while this value was 5.13 for the OT method.

However, in some cases the use of entropies for image thresholding has provided better results than even Otsu's method [43, 44].

### 3.4. Mulch Residue Analysis

The comparison of bare soil (where the mulch was gone) provided by each of the statistically reliable methods for the four types of mulches considered is shown in Table 4. These methods were OT, RC, and LE (Section 3.3, Table 3). ANOVA tables of the reference areas are also included to compare the results.

Table 4 is a comparison of mulch deterioration in actual field conditions, as measured by the percentage of bare soil area, by using different image thresholding methods. Data shown in Table 4 for the OT and RC methods in comparison with those obtained by ideal images are very similar, because ANOVA was significant in the three cases, and also because the mean values obtained in the deterioration of mulches by each method were quite similar. However, with the LE method, no significant differences between treatments (*P* > 0.05) were observed; the area of bare soil for mulch 3 was much overestimated (37.77% versus 17.65% for the reference image) and also altered the behavior of mulches 2 and 4 with respect to the reference image (Table 4). Therefore, we discard LE as an accurate thresholding method for soil and mulch image segmentation. As a result, we choose the automatic OT and RC methods as the best thresholding methods with very similar results to each other and also with respect to the reference images. In general terms, both are simple and well-known methods. The RC method has a slightly lower computational cost while the OT method has the advantage of being included in specialized software.

The mean values of rate of deterioration (% bare soil area) obtained by expert user (*A*_*R*_) and by the accurate thresholding methods (*A*_OT_, *A*_RC_) were, respectively, 50.90%, 50.60%, and 50.33% for mulch 1; 52.76%, 54.66%, and 54.10% for mulch 2; 17.65%, 19.62%, and 18.85% for mulch 3; 41.46%, 44.82 %, and 44.14 % for mulch 4.

The best thresholding methods obtained (OT and RC) indicate that mulch 3 (polyethylene) differs significantly from the others by presenting much less deterioration (a lower percentage of bare soil) than the biodegradable materials. As expected, these results highlight the low degradability of polyethylene in the soil and warn of the environmental problems this may cause.

## 4. Conclusions

The results obtained by four thresholding methods from color images containing soil and mulch were compared: Otsu (OT), Ridler-Calvard (RC), local entropy (LE) and manual thresholding (MT). Furthermore, deterioration of four mulch materials (polyethylene, two biodegradable plastics, and paper) was analyzed 100 days after implantation in the soil, through computation of the area of bare soil.

The following conclusions were reached.

- The problem of low-contrast color images in the field, with soil and mulch, can be solved by converting RGB to HSV color space and using the saturation plane histogram.
- Among the thresholding methods studied to obtain binary images, the most accurate ones with regard to the respective reference areas are Otsu and Ridler-Calvard.
- The percentage of missing mulch (soil area) has been automatically computed using binarized images.
- A hundred days after its implementation on the ground, biodegradable materials have deteriorated around 50%, well above the deterioration of polyethylene (below 20%).

The rate of deterioration of a mulch material, measured reliably and quickly in field conditions as we propose, is an important fact. It could help to better understand the overall behavior of the materials used as mulch. Therefore, the methods to obtain such data, as proposed in this study, will be useful to mulch manufacturers and farmers.

## Figures and Tables

**Table 1:** Thresholds and areas obtained after eliminating small objects. Thresholds: Otsu (*t*_OT_), Ridler-Calvard (*t*_RC_), manual thresholding (*t*_MT_), and local entropy (*t*_LE_). Soil areas by Otsu (*A*_OT_), Ridler-Calvard (*A*_RC_), manual thresholding (*A*_MT_), local entropy (*A*_LE_), and reference area (*A*_*R*_).

Image	Mulch∗	Block	Threshold				Area				
			*t*_OT_	*t*_RC_	*t*_MT_	*t*_LE_	*A*_OT_	*A*_RC_	*A*_MT_	*A*_LE_	*A*_*R*_
1	1	1	0.2902	0.2874	0.2468	0.5059	50.9052	49.4967	52.9680	39.8214	49.0100
2	1	2	0.2745	0.2717	0.2297	0.4667	46.5797	45.5628	50.4214	38.7190	55.7600
3	1	3	0.3451	0.3425	0.2158	0.5529	70.6396	70.8865	82.7414	61.2655	86.7600
4	1	4	0.2706	0.2677	0.2494	0.5098	46.4310	45.7977	47.7424	27.5502	39.7300
5	1	5	0.2353	0.2283	0.2390	0.4667	45.2831	46.4100	44.8275	9.6671	29.0600
6	1	6	0.2824	0.2795	0.2430	0.1804	43.7783	43.8106	45.0526	67.6796	45.0500
7	2	1	0.3255	0.3228	0.2691	0.5843	64.9501	63.4503	68.4051	43.6728	59.9000
8	2	2	0.2784	0.2756	0.2483	0.1922	46.8627	44.7348	47.8013	65.0698	45.5200
9	2	3	0.2745	0.2677	0.2499	0.1412	36.4270	35.7161	38.2370	57.5681	33.7800
10	2	4	0.3216	0.3189	0.2599	0.5608	68.6014	68.0766	71.7309	52.9240	71.0700
11	2	5	0.3137	0.3189	0.2897	0.3490	49.5972	50.1565	51.2083	52.3749	52.0200
12	2	6	0.3255	0.3228	0.2819	0.5529	61.5203	62.4738	66.2118	38.5616	54.2500
13	3	1	0.2588	0.2559	0.2616	0.0902	24.5614	24.6451	24.4695	45.8991	22.2100
14	3	2	0.2745	0.2677	0.2406	0.1804	31.0725	31.3840	32.8444	44.1760	23.8300
15	3	3	0.2667	0.2598	0.2400	0.1176	19.3475	18.8066	19.6754	45.0813	23.3600
16	3	4	0.2392	0.2362	0.2332	0.1412	28.6458	28.8813	29.0930	46.7529	29.2300
17	3	5	0.1569	0.1378	0.3919	0.9961	1.6047	1.9750	0.6406	0.0000	2.7600
18	3	6	0.2118	0.2047	0.4327	0.1529	12.4904	7.4138	0.8420	44.7230	4.5300
19	4	1	0.2353	0.2323	0.2297	0.4706	47.0578	47.1434	47.3097	6.9027	26.3000
20	4	2	0.3098	0.3071	0.2517	0.1333	42.9258	43.0054	45.3668	60.3160	42.6000
21	4	3	0.2980	0.2953	0.2773	0.1569	47.2435	46.5150	47.6497	66.6117	48.9300
22	4	4	0.2980	0.2953	0.2662	0.1608	46.5599	46.6459	47.7724	63.7267	47.6600
23	4	5	0.3059	0.2992	0.2622	0.2275	45.0941	45.9767	49.0865	59.3983	42.4800
24	4	6	0.2902	0.2874	0.2439	0.1490	40.0192	35.5562	41.6854	59.2816	40.7600

*Mulch 1: biodegradable (BD). Mulch 2: biodegradable (BD). Mulch 3: polyethylene (PE). Mulch 4: paper (PP).

**Table 2:** Computational time (in minutes) for the calculation of soil area in binary images obtained by several thresholding methods: Otsu (OT), Ridler-Calvard (RC), local entropy (LE), manual thresholding (MT), and reference area (*R*). This time has been estimated based on a set of 24 color images. The ratio has been calculated with respect to MT method.

	OT	RC	LE	MT	*R*
Time (minutes)	10.5	9	18	230	3200
Ratio (%)	4.5	3.9	7.8	100	1391

**Table 3:** Comparison of the soil area percentage values obtained by thresholding methods (Otsu (OT), Ridler-Calvard (RC), manual threshold (MT), and local entropy (LE)) with the reference area (*A*_*R*_). The comparison was analyzed with a paired 2-tailed Student's *t*-test and a paired 2-tailed Wilcoxon nonparametric test in case of LE.

Groups		Average			S.D. (Dif.)	*t*	*P* bilateral∗
G1	G2	Dif.	G1	G2			
*A*_*R*_	*A*_OT_	−1.73	40.69	42.42	7.42	−1.15	0.2637
*A*_*R*_	*A*_RC_	−1.17	40.69	41.86	7.61	−0.75	0.4611
**A**_**R**_	**A**_**MT**_	−3.22	40.69	43.91	6.53	−2.42	**0.0241**
*A*_*R*_	*A*_LE_	−5.05	40.69	45.73	19.23		0.0540

*In bold the pair of methods that present significant differences in the areas at level *α* = 0.05. S.D. (Dif.): standard deviation of the mean difference.

**Table 4:** Comparison of mulch deterioration by percentage of bare soil area (% of white pixels) ± standard error in binary images obtained by thresholding methods: Otsu (OT), Ridler-Calvard (RC), and local entropy (LE). The areas obtained based on the reference images (*A*_*R*_) are shown in the first column. Mulch materials: biodegradable (BD), polyethylene (PE), and paper (PP).

Mulch^∗,∗∗^	*A*_*R*_	*A*_OT_	*A*_RC_	*A*_LE_***
1	50.90 ± 8.06^a^	50.60 ± 4.12^a^	50.33 ± 4.18^a^	40.78 ± 8.74^a^
2	52.76 ± 5.17^a^	54.66 ± 5.05^a^	54.10 ± 5.14^a^	51.70 ± 3.88^a^
3	17.65 ± 4.54^b^	19.62 ± 4.52^b^	18.85 ± 4.85^b^	37.77 ± 7.56^a^
4	41.46 ± 3.30^a^	44.82 ± 1.16^a^	44.14 ± 1.82^a^	52.71 ± 9.24^a^

*P*-value	0.0019	0.0001	0.0002	0.3715

*Mulch 1: biodegradable (BD). Mulch 2: biodegradable (BD). Mulch 3: polyethylene (PE). Mulch 4: paper (PP).

∗∗100 days after mulch laying. ANOVA and Duncan test, *α* = 0.05. Different letters in the same column indicate significant differences between soil areas.

∗∗∗ *x* ^2^ transformation.

## Acronyms

*t*_MT_: Threshold obtained by manual thresholding
*t*_OT_: Threshold obtained by Otsu's method
*t*_RC_: Threshold obtained by Ridler-Calvard's method
*t*_LE_: Threshold obtained by local entropy method
*A*_*R*_: Reference area
*A*_MT_: Area obtained by manual thresholding
*A*_OT_: Area obtained by Otsu's method
*A*_RC_: Area obtained by Ridler-Calvard's method
*A*_LE_: Area obtained by local entropy method.
